# Effects of oxytocin administration on salivary sex hormone levels in autistic and neurotypical women

**DOI:** 10.1186/s13229-020-00326-5

**Published:** 2020-03-18

**Authors:** Tanya L. Procyshyn, Michael V. Lombardo, Meng-Chuan Lai, Bonnie Auyeung, Sarah K. Crockford, J Deakin, S. Soubramanian, A Sule, Simon Baron-Cohen, Richard A. I. Bethlehem

**Affiliations:** 1 Autism Research Centre, Department of Psychiatry, University of Cambridge, 18b Trumpington Road, Cambridge, CB2 8AH UK; 2 Laboratory for Autism and Neurodevelopmental Disorders, Center for Neuroscience and Cognitive Systems, Istituto Italiano di Tecnologia, Rovereto, Italy; 3 Centre for Addiction and Mental Health and The Hospital for Sick Children, Department of Psychiatry, University of Toronto, Toronto, ON Canada; 4 Department of Psychiatry, National Taiwan University Hospital and College of Medicine, Taipei, Taiwan; 5 Department of Psychology, School of Philosophy, Psychology and Language Sciences, University of Edinburgh, Edinburgh, UK; 6 Section of Theoretical and Applied Linguistics, University of Cambridge, Cambridge, UK; 7 Department of Psychiatry, University of Cambridge, Cambridge, UK; 8 Cambridgeshire and Peterborough NHS Foundation Trust, Cambridge, UK; 9 South West London and St. George’s Mental Health NHS Trust, London, UK; 10 Liaison Psychiatry Service, St Helier Hospital, Epsom and St Helier University Hospitals NHS Trust, Surrey, UK; 11 Behavioural and Clinical Neuroscience Institute, University of Cambridge, Cambridge, UK

**Keywords:** Oxytocin, Autism, Sex steroids, Testosterone, Oestradiol, Salivary hormone levels, Autistic women

## Abstract

**Background:**

Oxytocin administration, which may be of therapeutic value for individuals with social difficulties, is likely to affect endogenous levels of other socially relevant hormones. However, to date, the effects of oxytocin administration on endogenous hormones have only been examined in neurotypical individuals. The need to consider multi-hormone interactions is particularly warranted in oxytocin trials for autism due to evidence of irregularities in both oxytocin and sex steroid systems.

**Methods:**

In this double-blind cross-over study, saliva samples were collected from 16 autistic and 29 neurotypical women before and after intranasal administration of 24 IU oxytocin or placebo. Oestradiol, testosterone, and oxytocin levels were quantified in saliva samples. Participants also completed the Autism-Spectrum Quotient (AQ) and Empathy Quotient (EQ) questionnaires.

**Results:**

Distinct patterns of change in testosterone and oestradiol levels pre- to-post-administration were observed in autistic relative to neurotypical women (ANCOVA, *p* < 0.05 main effect of Group), controlling for sample collection time. The mean percent change oestradiol was + 8.8% for the autism group and − 13.0% for the neurotypical group (*t* = 1.81, *p* = 0.08), while the mean percent change testosterone was + 1.1% in the autism group and − 12.6% in the neurotypical group (*t* = 1.26, *p* = 0.22). In the oxytocin condition, the mean percent change oestradiol was + 12.6% in the autism group and − 6.9% in the neurotypical group (*t* = 1.78, *p* = 0.08), while the mean percent change testosterone was + 14.4% in the autism group and − 15.2% in the neurotypical group (*t* = 3.00, *p* = 0.006). Robust regression confirmed that group differences in percent change hormone levels were not driven by a small number of influential individuals. Baseline hormone levels did not differ between groups when considered individually. However, baseline testosterone relative to oestradiol (T:E2 ratio) was higher in autistic women (*p* = 0.023, Cohen’s *d* = 0.63), and this ratio correlated positively and negatively with AQ and EQ scores, respectively, in the combined sample.

**Limitations:**

Further studies with larger and more diverse autistic sample are warranted to confirm these effects.

**Conclusions:**

This study provides the first evidence that oxytocin influences endogenous testosterone levels in autistic individuals, with autistic women showing increases similar to previous reports of neurotypical men. These findings highlight the need to consider sex steroid hormones as a variable in future oxytocin trials.

## Background

The neuropeptide hormone oxytocin is known to modulate social behaviour across mammals including humans [1]. For this reason, oxytocin has attracted interest for its potential therapeutic applications in psychiatric conditions characterised by challenges with social interactions [2, 3]. Intranasal administration, a means of drug delivery to the brain [4], has become the standard method of assessing the effect of a single hormone such as oxytocin on behaviour. Despite recognition of the complexities of the neuroendocrinology underlying human social behaviour and cognition [5], studies rarely consider the potential influence of other socially relevant hormones when interpreting the results of oxytocin administration.

Short-term manipulation of oxytocin is likely to influence endogenous release of other hormones expected to exert their own effects on social behaviour [6]. For example, increased testosterone levels have been reported in men [7, 8], but not in women [9], who received oxytocin nasal spray versus placebo. Levels of arginine vasopressin, a neurohormone closely related to oxytocin, were found to increase in men and women following oxytocin administration [10]. Also in both sexes, the effects of oxytocin administration on parenting-related behaviours were found to depend on baseline endogenous testosterone levels [8, 11]. Notably, oxytocin and testosterone administration have shown opposing effects on various aspect of social behaviour in neurotypical populations and show opposite patterns of alteration in psychiatric conditions such as autism and schizophrenia [12], although such studies rarely assess multiple hormones within individuals.

While multi-hormone interplay and its relevance to human social behaviour are yet to be fully elucidated, interactions among oxytocin and sex steroid hormones are well documented in animal research. Using in vitro receptor autoradiography, testosterone has been shown to inhibit oxytocin receptor binding in the brains of male mice [13]. Castration increases the number of oxytocin immunoreactive neurons in the paraventricular nucleus of male mice, while castration plus testosterone implants decrease this number [14]. In rats, Leydig cells cultured with oxytocin or an oxytocin agonist produce higher levels of testosterone, and this increase in testosterone production is mediated by the oxytocin receptor [15]. Oestradiol treatment in ovariectomised rats alters the distribution of oxytocin immune-stained neurons and oxytocin levels in brain regions including the lateral septum, striatum, and amygdala [16]. Pre-treatment with oestradiol enhances the anxiolytic effect of oxytocin administration in female mice, possibly via enhancement of oxytocin binding density [17]. The likelihood of similar interactions between oxytocin and steroid hormones in humans is supported by an in vitro study of neuroblastoma cells demonstrating that the androgen receptor mediates downregulation of oxytocin gene expression [18]. Taken together, these studies suggest a broadly inhibitory relationship between testosterone and oxytocin and a broadly synergistic relationship between oestrogens and oxytocin, although these relationships may be further complicated by sex differences in steroid hormone levels and oxytocin receptor systems in the brain [19].

People with autism spectrum conditions (henceforth autism) are an important clinical group to inform the exploration of the interplay among hormones and its effects on social behaviour. The social and communication challenges that characterise autism [20] have been associated with lower endogenous oxytocin levels in autistic children [21–23]. Furthermore, several lines of evidence support that elevation of sex steroid hormones, including androgens and oestrogens, contributes to the likelihood of autism [24–27]. Nevertheless, interactions with sex steroid hormones have not been considered in previous trials assessing the effects of oxytocin administration on social cognition in autistic individuals [28].

To date, the effects of oxytocin administration on endogenous steroid hormone levels have been examined in neurotypical individuals. Weisman et al. [8] reported alterations in fathers’ salivary testosterone levels after oxytocin administration relative to placebo. Gossen et al. [7] reported alterations in serum testosterone and progesterone, but not in oestradiol, in neurotypical men after oxytocin administration. Increases in testosterone levels after central oxytocin administration have also been reported in male squirrel monkeys [29]. In a study of women, Holtfrerich et al. [9] found that post-administration salivary testosterone levels did not differ between oxytocin and control groups. However, whether changes in steroid hormone levels occur in individuals with an autism diagnosis following oxytocin administration remains untested. To better assess oxytocin’s potential as a therapeutic agent in autism, further study of the interplay among oxytocin, other socially relevant hormones, and biological sex is warranted.

To address these questions, we analysed salivary hormone levels in autistic and neurotypical women before and after intranasal administration of oxytocin or placebo. The aims of this study were threefold. The first is to assess changes in endogenous testosterone and oestradiol after oxytocin administration and whether these changes differed with autism diagnosis. Salivary oxytocin was also measured, which allowed us to confirm the manipulation of oxytocin and, given recent evidence that pre-treatment oxytocin level influences response to oxytocin in autistic children [30], include baseline oxytocin in the analyses. Second, the data were used to compare baseline hormone levels between autistic and neurotypical women. The third aim was to explore relationships between baseline hormone levels and two psychological variables linked to autism, namely the Autism-Spectrum Quotient (AQ) [31] and the Empathy Quotient (EQ) [32]. Given the literature on interactions between oxytocin and sex steroids, as well as the possibility of a hypermasculinised phenotype in autism [33, 34], we predict that oxytocin administration will promote testosterone decreases in neurotypical women but testosterone increases in autistic women—similar to previous reports for neurotypical men [7, 8]. Further, motivated by recent work reporting a role of oestrogens in autism [27], we predict a higher testosterone relative to oestradiol (T:E2 ratio) in autistic women and/or women with higher levels of autistic-like traits.

## Methods

### Participants

A total of 45 women aged 18–50 years participated in this study. Of these, 16 had a diagnosis of autistic disorder/childhood autism or Asperger’s disorder/syndrome based on DSM-IV or ICD-10 criteria (autism group), and 29 were neurotypical (neurotypical group). The two groups did not differ significantly in age or full IQ (Table 1). No participant had a history of psychotic disorders or substance use disorder; any genetic syndrome associated with autism; intellectual disability; epilepsy; hyperkinetic disorder; Tourette’s syndrome; or current or past use of anti-psychotic, glucocorticoid, psychostimulant, or antihypertensive drugs. Use of hormonal contraceptives and anti-depressants was permitted, as a significant proportion of the study population was expected to be taking such medication.

**Table 1:** Demographic characteristics, psychological questionnaire scores, and baseline hormone levels in the autism and neurotypical groups

	Autism	Neurotypical	*p-value*	*Cohen’s d*
***N***	16	29		
**Demographics**				
Age (years)	29.9 ± 8.4	27.2 ± 8.1	0.31	0.33
Full-IQ^1^	121.2 ± 16.4	114.6 ± 14.3	0.19	0.44
Hormonal contraceptive use (*n*)	0	8		
**Psychological variables**				
Autism-Spectrum Quotient (AQ)	37.1 ± 5.1	14.1 ± 7.5	< 0.01**	3.4
Empathy Quotient (EQ)	20.7 ± 11.7	54.6 ± 14.0	< 0.01**	− 2.5
**Baseline hormone levels**^**2**^				
Baseline oestradiol (pg/ml)	1.0 ± 0.3	1.2 ± 0.5	0.055	− 0.54
Baseline testosterone (pg/ml)	70.3 ± 24.9	66.4 ± 22.0	0.60	0.17
T:E2 ratio^3^	0.39 ± 0.63	− 0.22 ± 1.1	0.023 *	0.63
Baseline oxytocin (pg/ml)	3.1 ± 0.5	2.8 ± 0.6	0.064	0.57

Values are mean ± SD, unless otherwise specified

*p < 0.05, **p < 0.01

^1^Wechsler Abbreviated Scale of Intelligence

^2^Baseline hormone levels were calculated as the mean of the two pre-administration samples collected per participant

^3^Log-transformed and then normalised to have mean of 0 and standard deviation of 1

### Experimental design

Data were collected as part of a larger neuroimaging study [35] with a double-blind, placebo-controlled, within-subjects crossover design. Each participant was randomly assigned to receive placebo or oxytocin first. Normally-cycling participants self-reported date of last menstruation and were scheduled to participate during the early follicular stage in an effort to minimise differences in hormone levels between sessions. For participants taking hormonal contraceptives, the second session was scheduled a minimum of 1 week later to ensure complete wash-out. For most participants, the interval between sessions was 7–15 days; the maximum interval between sessions was 51 days.

Before participating in the experiment, participants underwent a short health screening (comprising heart and blood pressure measurement, inquiry about allergies, and pregnancy test) by a trained clinician and were deemed fit to participate. Participants received either a dose of 24 IU oxytocin (Syntocinon, Novartis, Switzerland) or placebo (prepared by Newcastle Specials Pharmacy Production Unit) and were instructed to self-administer three puffs to each nostril. Following administration, participants rested for 20 min before completing a neuroimaging session lasting approximately 1 h. The neuroimaging session comprised a resting state scan and three fMRI tasks. Participants completed the same series of neuroimaging tasks in each session.

Psychological self-report questionnaires were completed in advance of the experiment. The Autism-Spectrum Quotient (AQ) was used to quantify autistic-like traits [31], and the Empathy Quotient (EQ) was used to quantify the cognitive and affective domains of empathy [32].

### Saliva collection

Participants were instructed to refrain from consuming caffeinated beverages the day of the experiment and to refrain from consuming alcohol for 24 h prior to testing. Saliva samples were collected at three timepoints: (1) baseline (40 ± 20 min before administration), (2) shortly after intranasal administration of placebo or oxytocin (6 ± 4 min post-administration), and (3) after completion of the neuroimaging session described above (96 ± 19 min post-administration). The average interval between time 1 and 2 was thus 46 min, while the average interval between times 1 and 3 was 136 min. In total, six saliva samples (three in the oxytocin condition, three in the placebo condition) were collected per participant. Saliva samples were collected by passive drool and frozen immediately at − 80 °C until analysis.

### Salivary hormone analysis

Salivary hormone level analysis is a minimally invasive method commonly used in behavioural research [36]. Salivary oestradiol and testosterone were analysed using commercially-available enzyme-linked immunosorbent assay (ELISA) kits designed specifically for use with saliva (Salimetrics, USA). Assays were performed by the NIHR Cambridge Biomedical Research Centre, Core Biochemical Assay Laboratory. Oxytocin was quantified using a highly sensitive and specific radioimmunoassay (RIAgnosis, Germany); salivary oxytocin measured by this method correlates with oxytocin in cerebrospinal fluid [37]. Intra- and inter-assay variability was < 15% for oestradiol, < 12% for testosterone, and < 10% for oxytocin.

For two samples, the obtained oestradiol concentration was below the detection limit of the kit (< 0.1 pg/ml); as oestradiol values for other samples from those individuals were well above the detection limit, the low values were deemed invalid measurements and excluded from analysis. One baseline testosterone value and one baseline oestradiol value were identified as outliers (± 3 standard deviation (SD) of the mean). As these measurements appeared to be valid (i.e. other samples from the participant were at the higher range), these two values were winsorised (set at the highest value that was not an outlier) and included in the analysis. The number of samples used in the analyses was thus 90 per time point per hormone, with the exception of 88 samples for time point 2 for oestradiol.

Baseline hormone levels were calculated as the mean of the two samples collected before administration per participant. To assess the balance of sex steroid hormones, in line with previous research assessing multiple sex steroids in autism and published recommendations [27, 38], the baseline testosterone to baseline oestradiol ratio (T:E2 ratio) was computed as log(baseline testosterone) − log(baseline oestradiol) and then normalised to have a mean of 0 and standard deviation of 1. To explore post-administration changes in hormone levels, the percent change from time point 1 (to time points 2 (~ 6 min post-administration) and 3 (~ 96 min post-administration) was calculated as (final − initial)/initial).

### Statistical analysis

Data are reported as mean and standard deviation. Baseline hormone levels and psychological variables were compared between the autism and neurotypical groups using Welch’s *t* test. Changes in hormone levels over time within individuals were compared using paired *t* tests. Pre- to post-administration percent change in hormone levels between groups (autism or neurotypical) and drug condition (oxytocin or placebo) were assessed by analysis of covariance (ANCOVA), controlling for collection time of the baseline saliva sample and the interval (in minutes) between the initial and final sample. Because linear regression can be sensitive to small datasets, robust regression using iterated re-weighted least squares was also performed. Robust regression attempts to ignore or down-weight unusual data [39], offering further support that results are not driven by a small number of highly influential datapoints. Statistical analyses were performed using R version 3.6.2 [40]. Effect sizes were calculated using the “effsize” package, and the “MASS” package was used for robust regression.

## Results

### Participant characteristics and baseline hormone levels

A comparison of demographic characteristics, questionnaire scores, and baseline hormone levels between the autism and neurotypical groups is presented in Table 1. The two groups did not differ in terms of age, IQ, baseline oestradiol, baseline testosterone, or baseline oxytocin. The T:E2 ratio, however, was significantly different, with autistic women showing greater testosterone relative to oestrogen and neurotypical women showing the opposite pattern. Psychological variables also differed between groups in the expected direction, with autistic women having substantially higher AQ scores and lower EQ scores than neurotypical women. Baseline hormone levels were not related to age (*r* < 0.10, *p* > 0.50, all tests) and did not differ significantly between sessions (Welch’s *t* test, *p* > 0.45 all tests), or with hormonal contraceptive use (Welch’s *t* test, *p* > 0.11 all tests). Exclusion of the eight neurotypical women who reported taking hormonal contraceptives did not change these results (Supplementary Table S1).

**Fig. 1:**
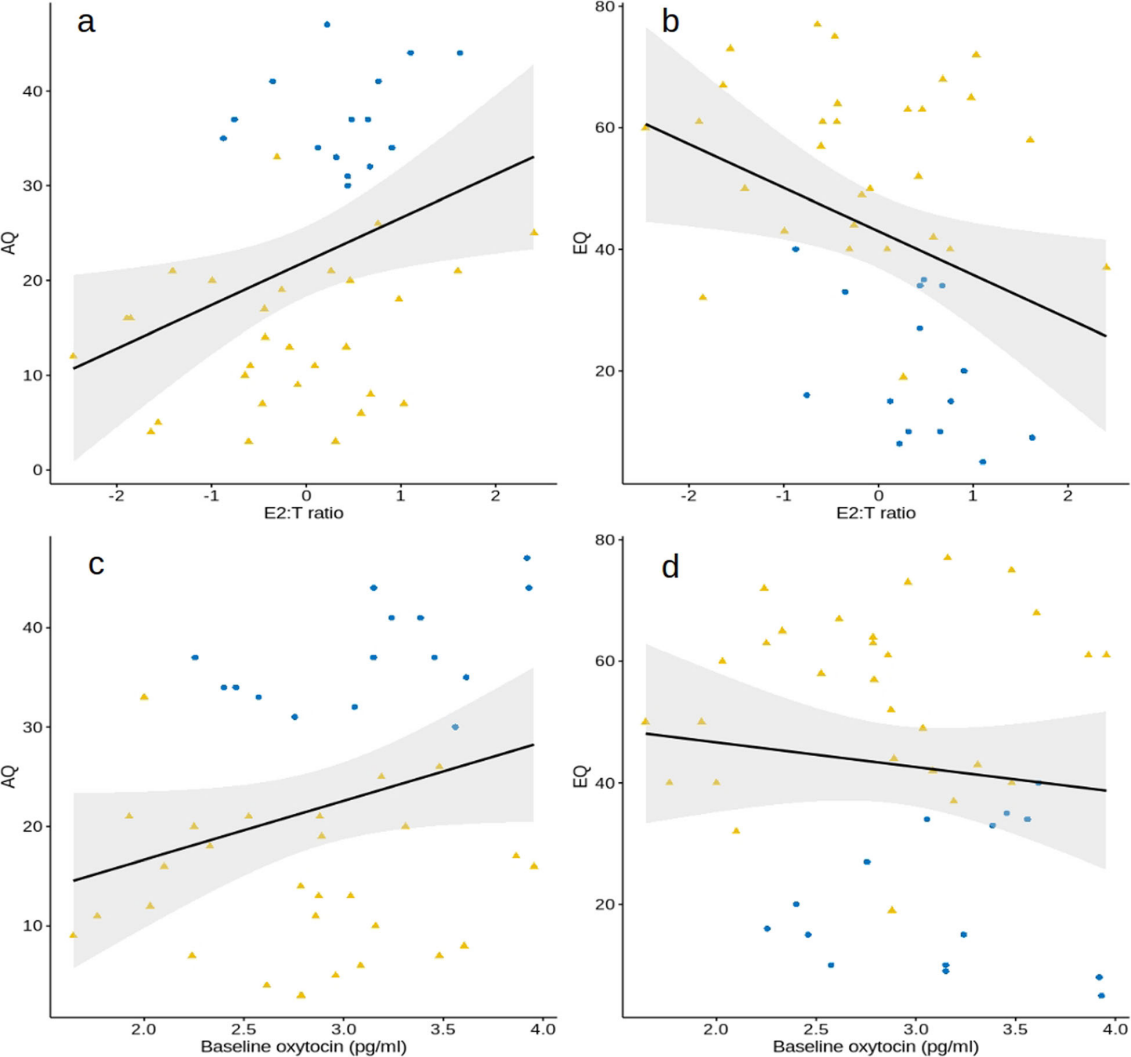
Correlations between hormonal and psychological variables: **a** T:E2 ratio (log-transformed and normalised ratio of baseline testosterone to oestradiol) and Autism-Spectrum Quotient (AQ) score; **b** ratio of baseline testosterone to oestradiol (log-transformed and normalised T:E2 ratio) and Empathy Quotient (EQ) score; **c** baseline oxytocin and AQ score; and **d** baseline oxytocin and EQ score. Regression line is indicated in black with 95% confidence intervals shaded in grey. Autistic and neurotypical women participants are indicated by darker blue circles and lighter yellow triangles, respectively

The relationships between baseline hormone levels and psychological traits are presented in Fig. 1. In the overall sample, greater baseline testosterone relative to oestradiol was positively correlated with AQ score (*r* = 0.36, *p* = 0.017), and negatively correlated with EQ score (*r* = − 0.35, *p* = 0.02). Baseline oxytocin showed a trend of a positive correlation with AQ score (*r* = 0.27, *p* = 0.067) but no relationship with EQ score (*r* = − 0.12, *p* = 0.45). Similar patterns were present when correlations were determined separately for the two groups (autism: *r* = 0.22, *p* = 0.44 for AQ and T:E2; *r* = − 0.44, *p* = 0.10 for EQ and T:E2; *r* = 0.51, *p* = 0.05 for oxytocin and AQ; *r* = 0.11, *p* = 0.69 for oxytocin and EQ; neurotypical: *r* = 0.25, *p* = 0.24 for AQ and T:E2; *r* = − 0.14, *p* = 0.43 for EQ and T:E2; *r* = − 0.05., *p* = 0.80 for oxytocin and AQ; *r* = 0.18, *p* = 0.35 for oxytocin and EQ), although statistical significance was not achieved with the smaller sample sizes.

### Pre- to post-administration changes in salivary hormone levels

**Fig. 2:**
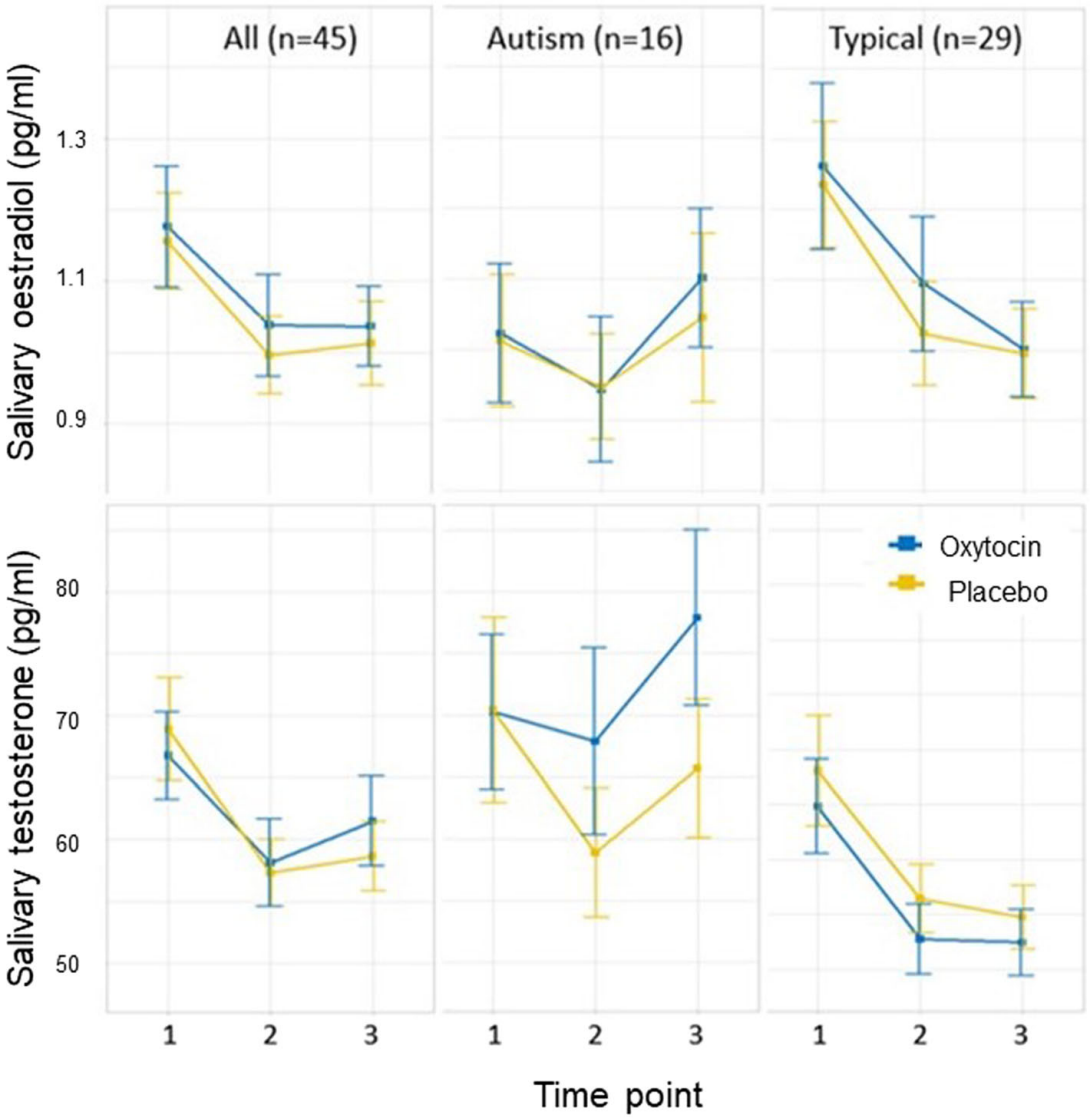
Mean salivary oestradiol levels (upper panels) and testosterone (lower panels) under the placebo and oxytocin conditions across the three measurement points (Time point 1 = before administration, 2 = ~ 6 min post-administration, 3 = ~96 min post-administration). Error bars indicate the standard error of the mean. From left to right, the panels show: (i) hormone levels separated by drug condition for all participants; (ii) hormone levels separated by drug condition for the autism group; and (iii) hormone levels separated by drug condition for the neurotypical group

Figure 2 presents the mean oestradiol and testosterone levels at each time point separated by group (autism and neurotypical) and drug condition (oxytocin and placebo) (see Supplementary Figures S2-S5 for individual level hormone levels). For the combined participants, paired tests indicated small but significant decreases (oestradiol: time 1 vs time 2, mean change of − 0.16 pg/ml, *p* < 0.001, time 1 vs time 3, mean change of − 0.14 pg/ml, *p* = 0.01; testosterone: time 1 vs time 2, mean change of − 10.1 pg/ml, *p* < 0.001, time 1 vs time 3, mean change of − 7.8 pg/ml, *p* < 0.01). Comparison of oxytocin levels across the three time points between drug conditions confirmed the effects of the manipulation (Supplementary Table S2).

**Fig. 3.**
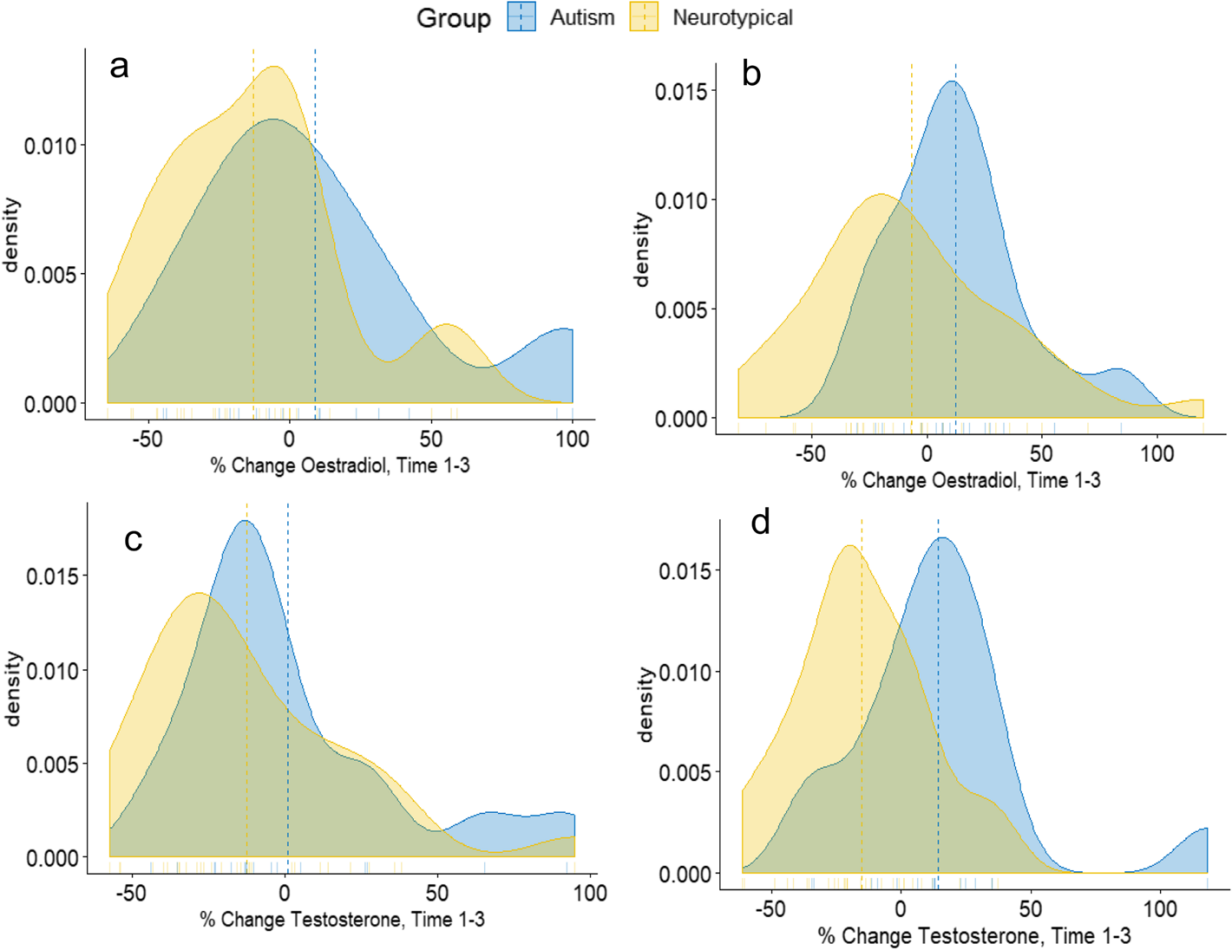
Density plots of percent change hormone levels from time 1 to 3 between autistic (indicated in blue) and neurotypical (indicated in yellow) groups: **a** percent change oestradiol under placebo condition; **b** percent change oestradiol under oxytocin condition; **c** percent change testosterone under placebo condition; **d** percent change testosterone under oxytocin condition. Dashed lines indicate the means for each group

Figure 3 presents the mean percent change oestradiol and testosterone from time point 1 to 3 separated by group (autism and neurotypical) and drug condition (oxytocin and placebo). ANCOVA revealed a significant group difference in percent change testosterone (F(1, 79) = 6.5, *p* = 0.01) and percent change oestradiol (F(1,79) = 4.3, *p* = 0.04), controlling for collection time of the baseline sample and the interval between time points (see Supplementary Tables S3 and S4 for full ANCOVA table). In the placebo condition, the mean percent change oestradiol was + 8.8% for the autism group and − 13.0% for the neurotypical group (*t* = 1.81, *p* = 0.08), while the mean percent change testosterone was + 1.1% in the autism group and − 12.6% in the neurotypical group (*t* = 1.26, *p* = 0.22). In the oxytocin condition, the mean percent change oestradiol was + 12.6% in the autism group and − 6.9% in the neurotypical group (*t* = 1.78, *p* = 0.08), while the mean percent change testosterone was + 14.4% in the autism group and − 15.2% in the neurotypical group (*t* = 3.00, *p* = 0.006, 95% confidence interval of difference = 9.3–50%). Exclusion of the eight neurotypical women taking hormonal contraceptives did not substantially change the results (Supplementary Tables S5, S6), nor was there an effect of baseline oxytocin when it was included in the analyses (Supplementary Tables S7, S8).

As the differences in percent change oestradiol between autistic and neurotypical women were not significant in the post hoc tests, they were not explored further. Thus, robust regression was performed only for analysis of percent change testosterone to ensure that the significant group difference was not driven by hormone level changes for a small number of highly influential individuals. Four observations (3 autistic participants, 1 neurotypical participant) were substantially down-weighted (< 0.60, Huber weight), which did not substantially change the statistics supporting the group difference. As further support for this group difference, two-sample tests for equality of proportions with continuity correction were performed. After oxytocin, testosterone levels increased in 11 of 16 autistic women but in only 8 of 29 neurotypical women (χ^2^ = 5.6, *p* = 0.018). By contrast, after placebo, the proportions of women showing increased testosterone levels between the two groups were nearly identical (5/16 autistic, 9/29 neurotypical, χ^2^ = 0.0, *p* = 1).

## Discussion

In the present study, we sought to identify interactions between oxytocin and steroid hormones that could potentially influence the outcomes of oxytocin administration in experimental and clinical settings. Given the underrepresentation of women in both autism research and endocrinology research, this work has made several important contributions to a sparse literature. First, on average, women in our study showed small but significant decreases in both salivary oestradiol and testosterone levels over time, which is consistent with expected diurnal rhythms of sex steroid hormone [41]. However, post hoc tests revealed that decreases in hormone levels over time were limited to neurotypical women, with autistic women instead showing average increases. Oxytocin enhanced the between-group difference in percent change testosterone, with the majority of autistic women (11 of 16) showing an increase and the majority of neurotypical women (21 of 29) showing a decrease over time. The pattern for autistic women is in line with the findings by Gossen et al. [7] that men’s testosterone levels increased over a time course of 210 min after oxytocin administration. In a study by Weisman et al. [8] on the effects of oxytocin administration on parent-child interactions, fathers’ testosterone levels decreased over time, but those who received oxytocin had significantly higher testosterone levels 40, 60, and 80 min after administration relative to the placebo condition. The pattern observed in autistic women is consistent with the idea of a masculinised phenotype [33, 34], as they showed sex hormone responses to oxytocin distinct from those of the neurotypical women in our study and more like the responses previously reported for neurotypical males. In the neurotypical women in our study, we see no evidence that oxytocin elevated testosterone levels relative to placebo—rather, testosterone levels were slightly higher under the placebo condition (time 2 56.4 ± 18.9 placebo, 52.7 ± 17.3 oxytocin; time 3 54.7 ± 15.6 placebo, 52.4 ± 16.2 oxytocin). In line with our findings for neurotypical women, Holtfrerich et al. [9] reported a small decrease in salivary testosterone for 29 women administered 24 IU oxytocin (oxytocin group: baseline = 25.05 ± 20.9 pg/ml, post-administration = 23.28 ± 12.7 pg/ml). Although the authors state that post-administration testosterone levels did not differ significantly between oxytocin and placebo groups, the control group comprised 28 different women with a lower baseline testosterone level (see Supplementary material of (9)).

In the present study, as testosterone levels tended to increase under both the placebo and oxytocin conditions in autistic women, this begs the question of why. Endogenous testosterone levels are reported to increase in contexts related to competition and mating [42], neither of which is applicable to our experimental context. One possibility is that autistic women experienced more stress during neuroimaging, as elevated testosterone levels have been reported in response to both social and physical stress [43]. Oxytocin—which is generally considered to have anxiolytic effects [44]—is thought to influence stress response via sex-specific effects on hypothalamic-pituitary-adrenal axis hormones [45]. However, in autism, oxytocin may enhance stress response, as shown in a rodent model of oxytocin-deficient female mice [46]. Grillon et al. [47] demonstrated that oxytocin can increase anxiety in humans, specifically defensive response to an unpredictable threat. Therefore, it is possible that testosterone increase in our autistic participants was related to stress and that oxytocin administration amplified stress reactivity. If oxytocin has anxiogenic effects in certain individuals, which may be indicated by increases in endogenous testosterone, they are unlikely to benefit from therapeutic interventions involving oxytocin.

Furthermore, our data presented a rare opportunity to compare hormone levels between autistic and neurotypical adult women. In our sample, we found no significant differences in baseline oestradiol, testosterone, or oxytocin when considered individually. The findings from previous studies comparing hormone levels between autistic and neurotypical adults are mixed. Ruta et al. [26] and Xu et al. [48] reported no difference in oestradiol levels in blood between a mixed sex sample of autistic adults (33 men, 25 women) vs. controls and 61 mothers of autistic children vs. control mothers, respectively. Ruta et al. [26] further reported no difference in testosterone or the testosterone to oestradiol ratio between autistic and neurotypical individuals. By contrast, Schwarz et al. [49] reported elevated testosterone levels in 23 women with Asperger’s syndrome relative to controls, while Xu et al. found elevated testosterone levels and reduced plasma oxytocin levels in mothers of autistic children relative to mothers of typically developing children [48]. While our study has the advantage of comparing hormone levels in two saliva samples, our study was underpowered to detect small differences due to the small sample size and high variability in salivary hormone measures.

Recent findings that medical conditions associated with elevated testosterone, such as polycystic ovary syndrome, are more common in autistic women [25, 50] supports potential roles of dysregulation of sex steroid hormone systems in autism, even if such differences are not reliably found in blood or saliva samples. Although it did not meet the threshold for statistical significance, the difference in testosterone levels between the autistic and neurotypical women in our study was in the predicted direction (see Table 1).

Interestingly, the ratio of testosterone to oestradiol differed between autistic and neurotypical women and was positively correlated with autistic-like traits (AQ score) and negatively correlated with self-reported empathy (EQ score) in our combined sample. Although the correlations are modest, they are broadly consistent with previous reports of associations between hormone measures and social traits. Prenatal testosterone levels, as measured in amniotic fluid, are positively and negatively correlated, respectively, with scores on the childhood versions of the AQ and EQ [51]. Adult women with elevated testosterone levels due to congenital adrenal hyperplasia are reported to have higher levels of autistic-like traits than controls [52], whereas girls with lower testosterone levels scored higher on cognitive empathy than girls with higher testosterone [53]. The aforementioned studies—like the present study—are based on small samples and thus warrant replication. Recent studies involving larger neurotypical samples have reported no relationship between salivary testosterone and AQ score in 67 young men [54] and no relationship between testosterone or oxytocin and AQ or EQ in 173 adults (94 women) [55]. Nevertheless, the findings of the present study suggest that consideration of multiple hormones within one pathway—such as oestradiol and testosterone—may better reflect relationships between hormone levels and social variables of relevance to autism. Indeed, a recent study has provided the first evidence that elevation of prenatal oestrogens contributes to the likelihood of autism in boys [27]. Whether prenatal oestrogens, or the ratio of androgens to oestrogens, contribute to autism likelihood in girls is yet to be tested.

## Limitations

This study has several limitations. First, as this study utilised saliva samples collected from participants during a neuroimaging experiment, participants were scheduled based on scanner availability rather than within a narrow time window typically used to assess hormones. Two time-related covariates were included in the analyses to account for expected diurnal variation. While decreases in testosterone levels later in the day are well-established in men, time of day may not affect salivary testosterone levels in women [56]. In our data, we saw no evidence of decreased hormone levels later in the day (see Figure S1). The menstrual cycle may also influence hormone levels, and participants were scheduled to participate in the experiment during the follicular stage to minimise differences between sessions. As menstrual stage was self-reported, this information may be unreliable. However, stage of menstrual cycle is not expected to greatly influence testosterone levels [56]. Second, due to the intervening neuroimaging session, saliva samples were limited to two time points post-administration. The optimal sampling time to test interactions between oxytocin and sex steroids is unclear. In previous studies of men [7, 8], elevated testosterone levels were reported 40 to 210 min after oxytocin administration. Our main measurement point (~ 96 min post-administration) was in the middle of this range. It is possible that the difference in testosterone levels between autistic and neurotypical women following oxytocin administration could vary with sampling time. Third, we cannot rule out the possibility that the neuroimaging experiment influenced hormone levels. However, as the same series of neuroimaging tasks was performed in each session, the effects of the task—if any—should be consistent across sessions. Lastly, as this is the first study to assess post-oxytocin administration hormone changes in autistic individuals and the sample size was relatively small, further studies are needed to confirm our main finding that neurotypical and autistic women show opposite patterns of sex steroid hormone changes in response to oxytocin. The effect of oxytocin administration on hormone levels in autistic men also warrants investigation. Future studies should strive to include individuals with more diverse phenotypes [57], as our autistic sample would be considered “high-functioning” and is not necessarily representative of the general autism phenotype.

Notably, not all autistic women showed increased testosterone levels and not all neurotypical women showed decreased testosterone levels after oxytocin administration. Whether individual changes in endogenous hormone levels are related to the effects of oxytocin on behaviour—particularly the social behaviours that oxytocin administration aims to enhance in clinical populations—is yet to be tested. In a recent oxytocin trial in autistic children, Parker et al. [30] found that low pre-treatment baseline oxytocin levels were predictive of improvement in social functioning with oxytocin. In an oxytocin administration study of neurotypical women, oxytocin administration was found to decrease response time to face stimuli but only among women with high endogenous testosterone levels [11]. Given the inconsistent findings of oxytocin clinical trials to date [58, 59], the possibility that baseline sex steroids or oxytocin-associated changes in sex steroids could serve as a biomarker of response may help to identify autistic people expected to benefit most from interventions involving oxytocin.

## Conclusions

This study provides further evidence that a single dose of oxytocin influences endogenous sex steroid levels, providing the first evidence of this effect in autistic individuals. Using a within-subjects, placebo-controlled design, opposite directional changes in salivary testosterone levels were found between autistic and neurotypical women, with autistic women showing increases in salivary testosterone levels, which is contra the expected diurnal pattern and consistent with previous reports in neurotypical men. Furthermore, we found that the ratio of testosterone to oestradiol differed between autistic and neurotypical women, and that this ratio correlated with self-reported autistic-like traits and empathy, consistent with predictions based on the social neuroendocrinology literature. Taken together, these findings highlight the need to consider current sex steroid hormone levels as a variable in future oxytocin trials in autism and to consider multiple sex steroids in studies of hormone system differences in autism.

## Supplementary information

**Additional file 1.** Supplementary materials

## Data Availability

The datasets used and/or analysed during the current study are available from the corresponding author on reasonable request.

## Abbreviations

ANCOVA: Analysis of covariance
AQ: Autism-Spectrum Quotient
ELISA: Enzyme-linked immunosorbent assay
EQ: Empathy Quotient
IU: International unit
T:E2: Ratio of baseline testosterone to oestradiol
