# Supplementary material for: Effects of oxytocin administration on salivary sex hormone levels in autistic and neurotypical women
Source: Mol Autism. 2020 Mar 18;11:20. doi: 10.1186/s13229-020-00326-5 (PMC7079504; doi:10.1186/s13229-020-00326-5)

**Table S1**. Comparison of demographic characteristics, psychological questionnaire scores, and baseline hormone levels in the Autism and Neurotypical groups excluding participants who reported taking hormonal contraceptives (n=8). Values are mean ± SD, unless otherwise specified.

|  | **Autism** | **Neurotypical** | ***p-value*** |
| --- | --- | --- | --- |
| **n** | 16 | 21 |  |
| **Demographics** |  |  |  |
| Age (years) | 29.9 ± 8.4 | 28.7 ± 9.0 | 0.55 |
| Full-IQ^1^ | 121.2 ± 20.0 | 113.3 ± 14.7 | 0.14 |
| Autism-Spectrum Quotient (AQ) | 37.1 ± 5.1 | 13.8 ± 8.1 | < 0.01** |
| **Baseline hormone levels^2^** |  |  |  |
| Baseline oestradiol (pg/ml) | 1.0 ± 0.3 | 1.2 ± 0.5 | 0.19 |
| Baseline testosterone (pg/ml) | 70.3 ± 24.9 | 69.4 ± 21.4 | 0.91 |
| Baseline oxytocin (pg/ml) | 3.1 ± 0.5 | 2.8 ± 0.6 | 0.16 |

^1^ Wechsler Abbreviated Scale of Intelligence ^2^ Baseline hormone levels were calculated as the mean of the two pre-administration samples collected per participant.

* p < 0.05, ** p < 0.01

**Table S2**. Mean salivary oxytocin levels (pg/ml) before and after administration of oxytocin or placebo.

|  | **Time 1**  **(~40 min  pre-administration)** | **Time 2**  **(~6 min  post-administration)** | **Time 3**  **(~96 min  post-administration)** |
| --- | --- | --- | --- |
| **Placebo** (n = 45) | 2.8 ± 0.8 | 2.7 ± 0.9 | 2.7 ± 0.8 |
| **Oxytocin** (n = 45) | 3.0 ± 0.9 | 112.4 ± 16.8 | 39.2 ± 19.0 |

**Table S3**. Full ANCOVA results for comparison of %change testosterone from time point 1-3 between groups and drug conditions, controlling for two time-related variables.

|  | **Sum Sq**^1^ | **Df** | **F value** | **Pr(>F)** |
| --- | --- | --- | --- | --- |
| Time 1 | 0.2049 | 1 | 2.0073 | 0.16048 |
| Interval time 1-3 | 0.1142 | 1 | 1.1185 | 0.29347 |
| Group | 0.6660 | 1 | 6.5229 | 0.01257 * |
| Drug condition | 0.0133 | 1 | 0.1305 | 0.71883 |
| Group x Drug condition | 0.1601 | 1 | 1.5678 | 0.21422 |
| Residuals | 8.0658 | 79 |  |  |

* p < 0.05
^1^ Type II sum of squares.

**Table S4**. Full ANCOVA results for comparison of %change oestradiol from time point 1-3 between groups and drug conditions, controlling for two time-related variables.

|  | **Sum Sq^1^** | **Df** | **F value** | **Pr(>F)** |
| --- | --- | --- | --- | --- |
| Time 1 | 0.1172 | 1 | 0.7920 | 0.37620 |
| Interval time 1-3 | 0.0086 | 1 | 0.0582 | 0.81001 |
| Group | 0.6313 | 1 | 4.2668 | 0.04214 * |
| Drug condition | 0.0534 | 1 | 0.3611 | 0.54961 |
| Group x Drug condition | 0.0247 | 1 | 0.1667 | 0.68415 |
| Residuals | 11.6877 | 79 |  |  |

* p < 0.05
^1^ Type II sum of squares.

**Table S5**. Full ANCOVA results for comparison of %change testosterone from time point 1-3 between groups and drug conditions, controlling for two time-related variables, excluding 8 neurotypical women taking hormonal contraceptives.

|  | **Sum Sq** | **Df** | **F value** | **Pr(>F)** |
| --- | --- | --- | --- | --- |
| Time 1 | 0.0254 | 1 | 0.2423 | 0.62427 |
| Interval time 1-3 | 0.0278 | 1 | 0.2655 | 0.60819 |
| Group | 0.4043 | 1 | 3.8633 | 0.05376 . |
| Drug condition | 0.0024 | 1 | 0.0230 | 0.87983 |
| Groupx Drug condition | 0.2493 | 1 | 2.3821 | 0.12774 |
| Residuals | 6.5935 | 63 |  |  |

. p < 0.10
^1^ Type II sum of squares.

**Table S6**. Full ANCOVA results for comparison of %change oestradiol from time point 1-3 between groups and drug conditions, controlling for two time-related variables, excluding 8 neurotypical women taking hormonal contraceptives.

|  | **Sum Sq** | **Df** | **F value** | **Pr(>F)** |
| --- | --- | --- | --- | --- |
| Time 1 | 0.0000 | 1 | 0.0003 | 0.98565 |
| Interval time 1-3 | 0.0996 | 1 | 0.6601 | 0.41959 |
| Group | 0.4348 | 1 | 2.8828 | 0.09446 . |
| Drug condition | 0.0465 | 1 | 0.3082 | 0.58078 |
| Group x Drug condition | 0.0198 | 1 | 0.1315 | 0.71812 |
| Residuals | 9.5017 | 63 |  |  |

. p < 0.10
^1^ Type II sum of squares.

**Table S7**. Full ANCOVA results for comparison of %change testosterone from time point 1-3 between groups and drug conditions including baseline oxytocin level.

|  | **Sum Sq** | **Df** | **F value** | **Pr(>F)** |
| --- | --- | --- | --- | --- |
| Time 1 | 0.1013 | 1 | 0.9828 | 0.32470 |
| Interval time 1-3 | 0.1145 | 1 | 1.1109 | 0.29527 |
| Baseline oxytocin | 0.2281 | 1 | 2.2141 | 0.14095 |
| Group | 0.5960 | 1 | 5.7848 | 0.01863 * |
| Drug condition | 0.0139 | 1 | 0.1353 | 0.71406 |
| Oxytocin x Group | 0.0160 | 1 | 0.1549 | 0.69501 |
| Oxytocin x Drug condition | 0.0148 | 1 | 0.1441 | 0.70533 |
| Group x Drug condition | 0.1736 | 1 | 1.6846 | 0.19829 |
| Oxytocin x Group x Drug condition | 0.0796 | 1 | 0.7728 | 0.38217 |
| Residuals | 7.7269 | 75 |  |  |

* p < 0.05
^1^ Type II sum of squares.

**Table S8**. Full ANCOVA results for comparison of %change oestradiol from time point 1-3 between groups and drug conditions including baseline oxytocin level.

|  | **Sum Sq** | **Df** | **F value** | **Pr(>F)** |
| --- | --- | --- | --- | --- |
| Time 1 | 0.1465 | 1 | 0.9561 | 0.33132 |
| Interval time 1-3 | 0.0023 | 1 | 0.0153 | 0.90186 |
| Oxytocin | 0.0395 | 1 | 0.2580 | 0.61301 |
| Group | 0.5698 | 1 | 3.7179 | 0.05762 . |
| Drug condition | 0.0957 | 1 | 0.6243 | 0.43196 |
| Oxytocin x Group | 0.1149 | 1 | 0.7500 | 0.38925 |
| Oxytocin x Drug condition | 0.0125 | 1 | 0.0814 | 0.77617 |
| Group x Drug condition | 0.0040 | 1 | 0.0260 | 0.87227 |
| Oxytocin x Group x Drug condition | 0.0265 | 1 | 0.1726 | 0.67900 |
| Residuals | 11.4953 | 75 |  |  |

. p < 0.10
^1^ Type II sum of squares.

**Figure S1.** Correlation between time of sample collection (in minutes) and pre-administration oestradiol (left) and testosterone (right) levels.

**
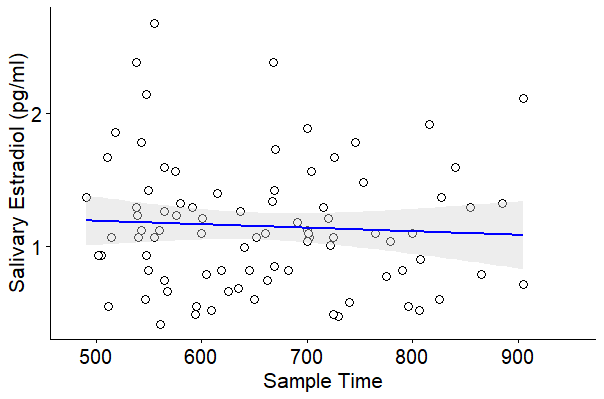
**

**
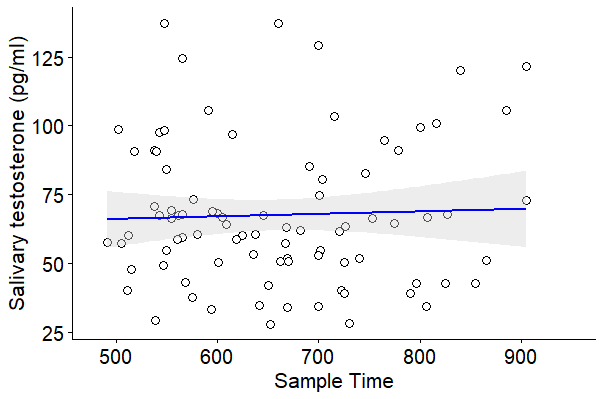
**

**Figure S2.** Salivary oestradiol levels across the three time points (1 = baseline, ~40 min before administration; 2 = ~6 min post-administration; 3 = ~96 min post-administration) for each participant under oxytocin (left) and placebo (right) drug conditions. Autistic participants are indicated in blue while Neurotypical participants are in yellow.


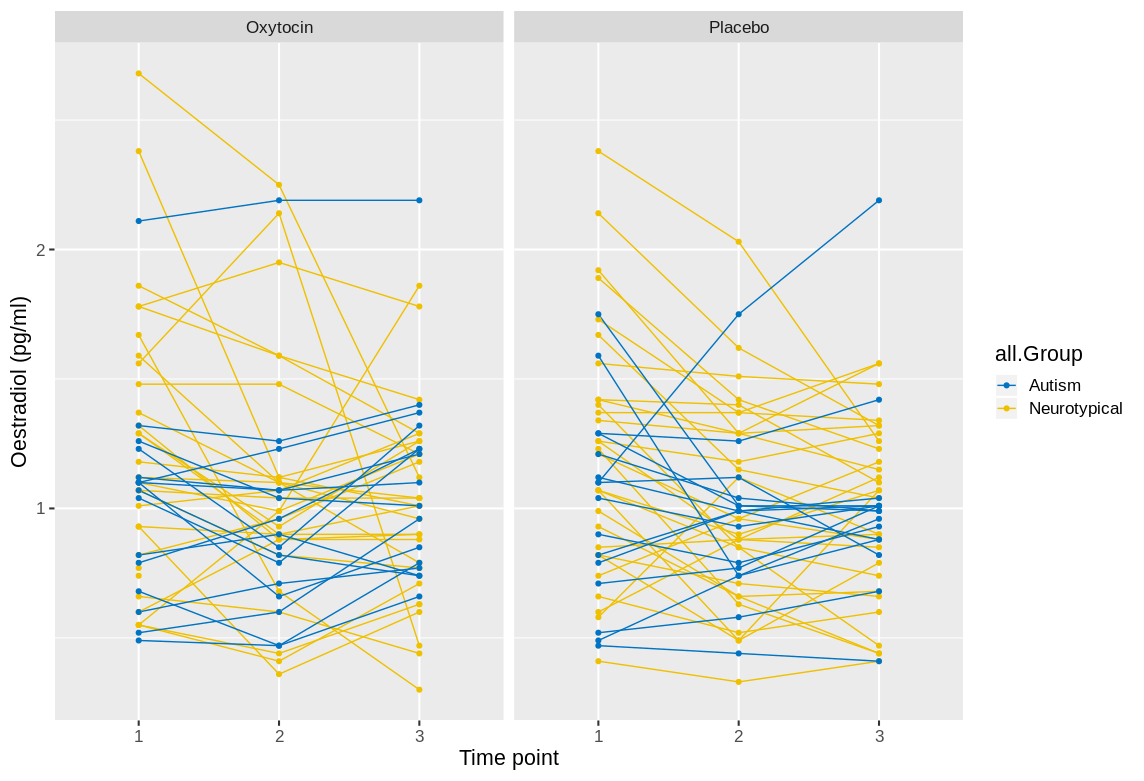


**Figure S3.** Salivary testosterone levels across the three time points (1 = baseline, 2 = ~6 min post-administration, 3 = ~96 min post-administration) each participant under oxytocin (left) and placebo (right) drug conditions. Autistic participants are indicated in blue while Neurotypical participants are in yellow.


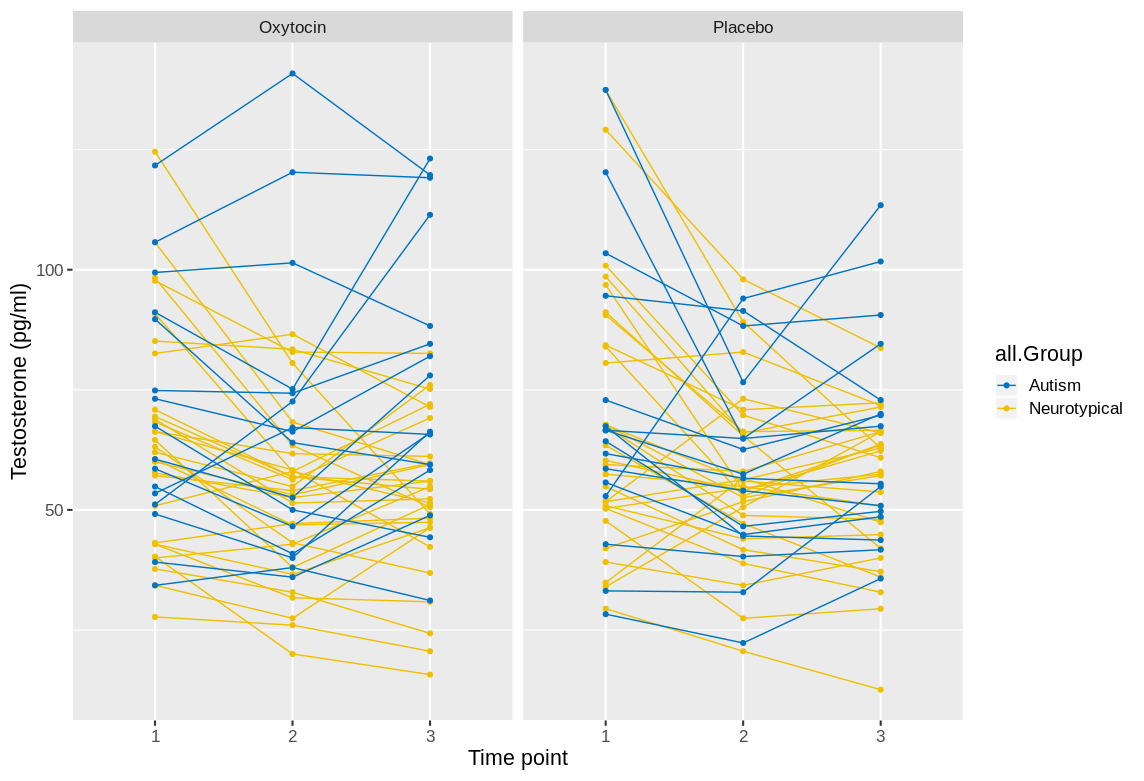


**Figure S4.** Ratio of salivary testosterone to oestradiol across the three time points (1 = baseline, 2 = ~6 min post-administration, 3 = ~96 min post-administration) each participant under oxytocin (left) and placebo (right) drug conditions. Autistic participants are indicated in blue while Neurotypical participants are in yellow.


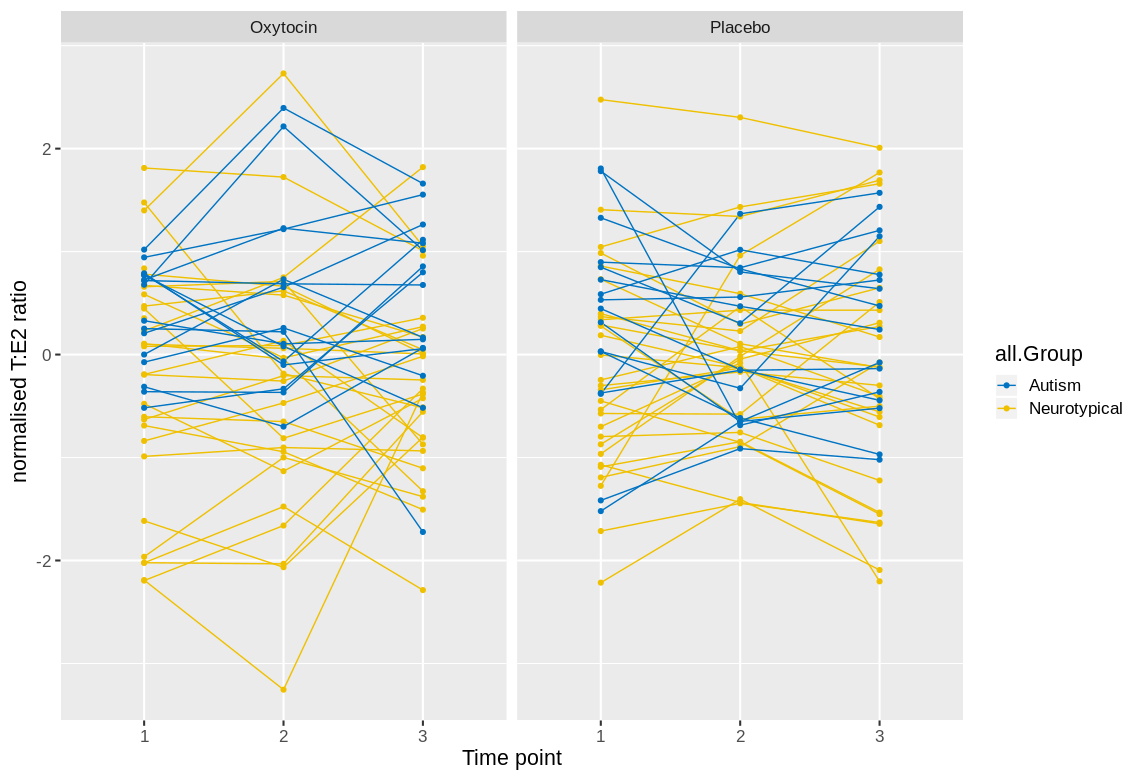

Supplement: Supplementary file 1 — Additional file 1. Supplementary materials [file 13229_2020_326_MOESM1_ESM.doc]
